# Prevalence and factors associated with hepatitis B surface antigen (HBsAg) positivity in Bugoye Sub-County, Western Uganda

**DOI:** 10.1186/s12982-026-02653-z

**Published:** 2026-08-12

**Authors:** Brian Turigye, Edgar M. Mulogo, Peyton Thompson, Morgan Camille, Varun Goel, Emmanuel Baguma, Khadijah Bhatti, Ross M. Boyce

**Affiliations:** 1 Department of Community Health, Faculty of Medicine, Mbarara University of Science and Technology, Mbarara, Uganda; 2 Department of Paediatrics, Division of Infectious Diseases, University of North Carolina at Chapel Hill, Chapel Hill, NC 27599 USA; 3 Department of Epidemiology, Gillings School of Global Public Health, University of North Carolina at Chapel Hill, Chapel Hill, NC 27599 USA; 4 Department of Geography, University of South Carolina, Columbia, SC 29208 USA; 5 Department of Medicine, University of North Carolina at Chapel Hill, Chapel Hill, NC 27599 USA; 6 Institute for Global Health and Infectious Diseases, University of North Carolina at Chapel Hill, Chapel Hill, NC 27599 USA

**Keywords:** Hepatitis B, Prevalence, Vaccination

## Abstract

**Background:**

Hepatitis B virus (HBV) infection is a major cause of global morbidity and mortality. National serosurveys suggest that Uganda has a prevalence of 4.3%of chronic HBV infection. However, there is limited information regarding the potential determinants for infection.

**Methods:**

Secondary data from a population-based household survey in the Kasese District were used to estimate HBsAg positivity. From these results, two villages were purposefully selected (i.e., low and high prevalence) for a population-representative household survey and a descriptive cross-sectional study. Descriptive analyses were conducted to characterise HBsAg positivity according to demographic, social-behavioural, clinical, and health-system factors, and spatial kernel density analysis and scan statistics were used to detect high-risk areas.

**Results:**

A total of 2,067 adults were tested for HBV, of whom 93 were HBsAg-positive, giving an overall seroprevalence of 4.5% (95% CI: 3.6–5.5). In the two selected villages, 286 participants were enrolled, including 125 individuals aged ≥ 15 years and 161 children aged < 15 years. HBsAg positivity was detected in 6 of 125 adults (4.8%) and in no children. Descriptive comparisons suggested that HBsAg-positive participants more frequently reported a history of HBV testing, blood transfusion, and receipt of parenteral medications. A significant spatial cluster of elevated HBV risk was identified in the central region of the study area (RR = 5.22, *p* = 0.0266).

**Conclusions:**

The prevalence of HBsAg is highly variable between villages in rural western Uganda. Infection was exclusively found in adults, which suggests. This suggests the benefit of hepatitis vaccination among children and could suggest that vertical and early-life transmission may be less of a risk factor. In adults with HBV, we observed a higher frequency of prior healthcare-associated exposures, which merits further investigation into practices intended to prevent transmission of blood-borne pathogens.

## Background

Viral hepatitis remains a major global public health challenge, causing an estimated 1.3 million deaths in 2022 and approximately 304 million people living with chronic hepatitis B or C infection worldwide [1]. Most deaths are the result of chronic liver disease (i.e., cirrhosis) and hepatocellular carcinoma, which usually develop years after initial infection. Hepatitis B virus (HBV) accounts for approximately half of global mortality associated with viral hepatitis [2]. Nearly 300 million people are estimated to be HBV surface antigen (HBsAg) positive - indicative of exposure - with the highest prevalence in the Western Pacific and African regions [3]. Many hepatitis B infections in sub-Saharan Africa are acquired early in life through either vertical transmission (mother-to-child transmission during pregnancy, delivery, or the perinatal period) or horizontal transmission through close household contact with infected family members and other children [4]. While prevention of mother-to-child transmission is a major public health priority, horizontal transmission during early childhood remains an important route of infection in many high-endemic settings in Africa [4, 5]. Infection acquired during infancy or early childhood carries a high risk of progression to chronic hepatitis B infection and its long-term complications, including cirrhosis and hepatocellular carcinoma [4]. Because chronic HBV infection is often asymptomatic, many infected individuals remain unaware of their status and therefore do not benefit from available prevention and treatment interventions while continuing to contribute to onward transmission [6].

Sub-Saharan Africa (SSA) has the highest prevalence of HBV, with more than 8% of the adult population estimated to be chronically infected [7]. In Uganda, the public health burden of HBV is not as well studied as other communicable diseases, but the prevalence of HBV is categorised as high [8]. According to the 2016–2017 Uganda Population-based HIV Impact Assessment (UPHIA), the overall prevalence of HBsAg among Ugandan adults was 4.3%, but this estimate masks the substantial variability between regions. For example, the northern region of the country had the highest prevalence (4.6%), while the southwest region had the lowest (0.8%) [9].

Potential drivers of transmission in Uganda, largely derived from studies conducted in northern regions, include low perceived risk of infection, delayed administration of the HBV vaccine, and delayed or absent HBV screening for mothers [10, 11]. While screening for HBsAg at the first antenatal visit is recommended as part of routine care [10], lower-level clinics frequently experience stockouts of rapid tests. Furthermore, even when rapid tests are available, women who screen positive must seek additional evaluation, typically at higher-level, often more distant facilities, prior to initiating antiviral prophylaxis [12, 13]. This places a substantial financial burden on the patient, not only for transportation, but also for laboratory testing, which may not be offered in the public health system.

There are also relatively unique cultural practices that may contribute to infection, such as the clustering of families, traditional practices such as body piercing that involve the use of sharp objects, and the widespread utilisation of traditional birth attendants [10, 14]. Lastly, many of the northern districts have been affected by conflict and large-scale population displacements, which have destabilised the health system [15].

However, much less is known about HBV in other regions of the country. Notably, the Kasese District, which is located in western Uganda, is one of eleven districts with the highest prevalence of HBV per the Ministry of Health data [16]. To our knowledge, however, there are no prior studies that have explored potential drivers of HBV transmission in this area. Kasese district is largely occupied by the Bantu Ethnic group that has traditional cultural practices distinct from the northern part of Uganda, which is largely occupied by the Nilotic. These areas also have different geopolitical and economic contexts [17–19]. Therefore, the objective of this study was to establish the prevalence of HBsAg in this rural highland region and identify risk factors for infection.

## Methods

### Study site

The Bugoye sub-county, located in the Kasese District of Western Uganda, comprises 35 villages, spanning a rural, highland area of approximately 55 km^2^. The population of the sub-county is 50,249, approximately one-quarter of whom are children under five years of age [20]. The geography of the sub-county is highly varied and characterised by deep river valleys and steep hillsides with elevations up to 2,500 m [21].

The sub-county’s primary public health facility is the Bugoye Level III Health Centre (BHC). BHC is comprised of a 25-bed inpatient ward, where patients can receive intravenous medications, a busy outpatient clinic that evaluates 60–80 patients per day, a maternity ward, and a small laboratory capable of performing point-of-care tests for diseases such as malaria and HIV. There are also level II health centres in each of the six parishes that offer basic outpatient services, including routine vaccination, and one private-not-for-profit level III health centre [22, 23].

### Study design

**Fig. 1:**
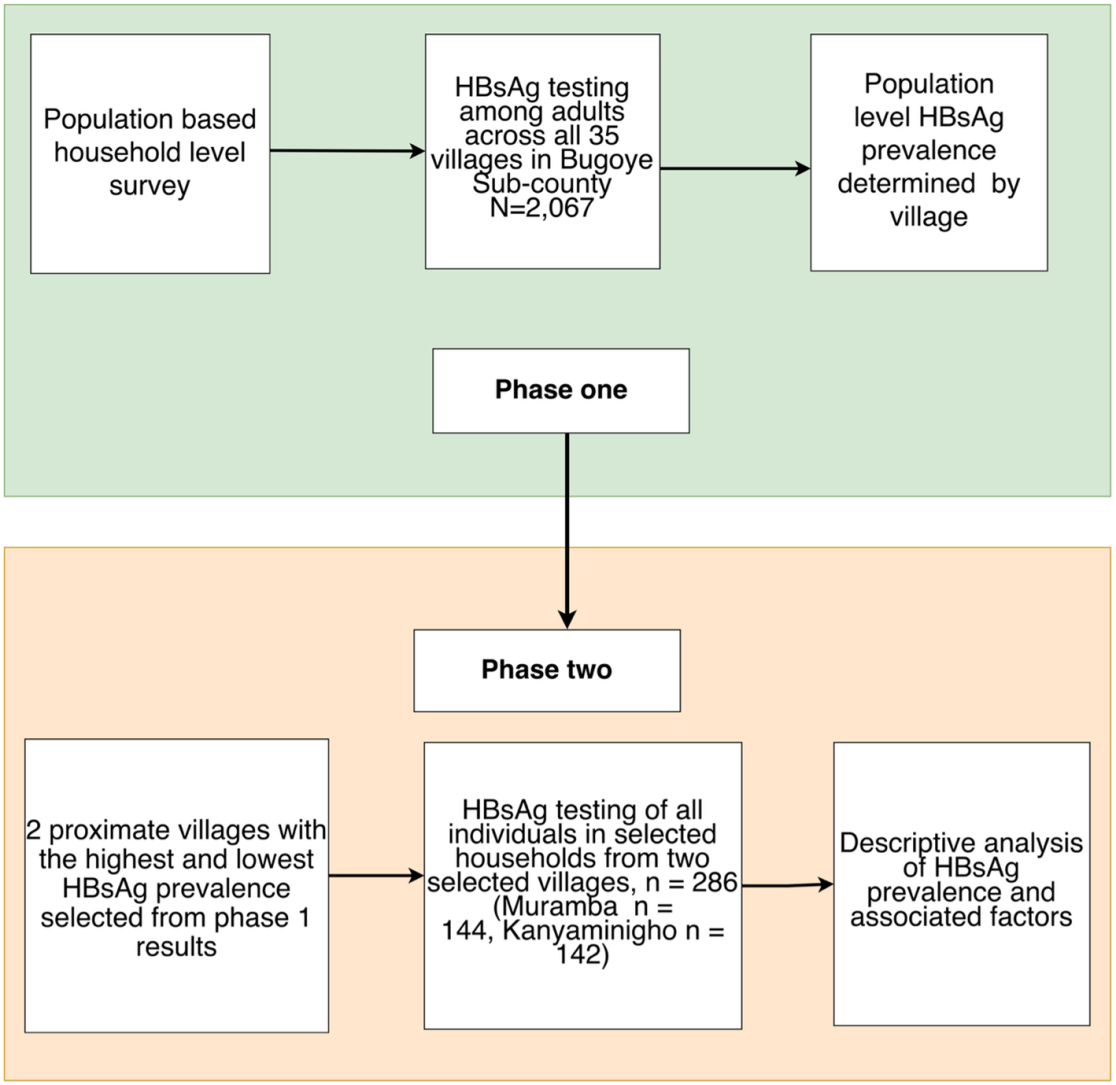
Study flow diagram showing HBsAg testing and village selection across the two study phases. Phase 1 involved a population-based household survey across all 35 villages in Bugoye Sub-county. Based on village-level HBsAg prevalence patterns, two geographically proximate villages with contrasting prevalence levels were purposively selected for Phase 2, during which all eligible household members in selected households were tested for HBsAg

The study was conducted in two distinct phases **(**Fig. 1**).** In Phase 1, we performed a cross-sectional survey of all households in the sub-county, carried out in conjunction with an ongoing study on malaria. The methods of this survey have been previously described [24]. In brief, we employed a stratified random sample design with the village being the unit of stratification. Starting from their own home, Community Health workers (CHWs) guided study staff in a clockwise direction to the nearest household and recorded the household location using a Global Positioning System (GPS)n device. In this phase, testing for HBV was offered to the adult present in the household. If the adult agreed to testing, study staff performed a RDT detecting hepatitis B surface antigen (HBsAg) (Standard Q HBsAg, SD Biosensor, Republic of Korea), the results of which were provided to the participant. If the index adult tested positive, testing was offered to all other members of the household. Individuals with positive HBsAg results were subsequently referred to the nearest level III health centre for further evaluation.

In Phase 2, we used the results of the household survey to purposefully select two villages that were (i) at extremes of HBsAg prevalence (i.e., relatively high vs. low), but (ii) located in close proximity. Selecting villages at the extremes of prevalence while in similar geographic locations and access to health services was intended to minimise confounding by geography and healthcare access, thereby allowing a more focused exploration of behavioural, social, and household-level factors associated with HBsAg positivity. We hypothesised that specific behaviours, rather than reduced access to healthcare services (e.g., facility-based delivery, immunisation), were the primary risk factors for HBsAg positivity. We purposively selected the village of Muramba 2, where the initial estimate of seroprevalence was 11.3% (6 of 53, 95% confidence interval [CI] 8.2–15.4%), as the high-prevalence village, while the village of Kanyaminigho, in which no adults (0 of 58) tested positive, was chosen as the low-prevalence village. The sample size in Phase 2 was determined pragmatically based on the number of households available in the two selected villages.

Households were selected using systematic sampling. With the basic sampling unit of a household, a sampling interval was determined based on the number of households in each of the selected villages. The list of households was obtained through the Village Health Teams (VHTs). The research assistant visited the household next to the VHT and then moved in a clockwise direction, visiting the households while skipping one household before going to the next until the required number of households had been surveyed. After consent was obtained, HBsAg rapid testing was performed for all household members present. Data were collected through direct interviews from each household member above 15 years of age using an instrument modified from a similar study [25], and a separate questionnaire documenting household factors (e.g., nature of the dwelling, average family income, and average family size), which was completed by the head of household. Questionnaires were administered prior to HBsAg testing.

### Data management and statistical analysis

Data from household surveys were recorded in REDCap using mobile devices with wireless internet connectivity [26]. Collected data were exported to STATA version 14 (Stata Corp, X) for cleaning and analysis [27].

In Phase 1, HBV prevalence was estimated as the proportion of individuals who had a positive HBsAg RDT result with 95% confidence intervals. Weighted estimates were generated using the svyset command in Stata, which accounted for the estimated probability of selection for each household, sample stratification, and the finite population correction (FPC) factors. Village population estimates were obtained from the most recent CHW census and were used to determine sampling weights and FPC factors. To visualise geographic variation in HBV risk, we estimated kernel-smoothed spatial relative risk surfaces across the study area to generate a heat map. Kernel smoothing is a routinely used non-parametric approach to estimate the relative risk of a disease based on point locations of disease cases and controls over a geographic region [28, 29]. We then used a spatial relative risk to identify areas where HBsAg prevalence was relatively higher (i.e., clusters) than other areas [30, 31].

We also examined potential differences in demographic characteristics, individual behaviours, and clinical and health system characteristics that might explain the disparate prevalence of HBsAg positivity between the high (Muramba 2) and low (Kanyaminigho) prevalence villages using phase 2 data; we assessed the difference in these characteristics across villages of residence using the Pearson chi-square statistic.

To explore the distribution of HBsAg positivity and associated factors, descriptive comparisons were conducted across demographic, social-behavioural, clinical, and health system characteristics among participants aged ≥ 15 years. Characteristics are presented according to HBsAg status using frequencies and percentages. Owing to the small number of HBsAg-positive cases, regression analyses were not considered sufficiently robust for inference and were therefore omitted.

### Ethical considerations

Ethical approval of the study was provided by the institutional review boards of the University of North Carolina at Chapel Hill (19-1094), the Mbarara University of Science and Technology (06/03–19), and the Uganda National Council for Science and Technology (HS 2628).

## Results

From January 8 to March 11, 2020, field staff surveyed a total of 2,067 households, representing 31.8% of all households in the sub-county. A total of 2,067 (94.38%) adult caregivers consented to testing for HBV, the majority (*n* = 1,283, 62.07%) of whom were mothers. The overall seroprevalence of HBsAg was 93/2067 (4.5%, 95% CI 3.6–5.5), but when disaggregated by villages, the seroprevalence varied markedly, ranging from *n* = 0 of 58 (0.0%) in Kanyamingho to *n* = 6 of 53 (11.3%) in Muramba 2. In six (6) of the 35 villages, including Kanyamingho, no individuals tested positive for HBsAg (Table 1).

**Table 1:** Prevalence of HBsAg in Bugoye subcounty by parish and village

Village	Adults tested	HBsAg RDT positivity		95% Confidence Interval
		*n*	(%)	
Overral	2067	93	(4.5)	[3.55,5.48]
Bugoye Parish				
Bugoye	69	2	(2.9)	[0.353, 10.081]
Kanyamingho #	58	0	(0)	[0, 6.162]
Kisamba 1	54	5	(9.3)	[3.075, 20.300]
Kisamba 2	55	3	(5.5)	[1.139, 15.123]
Muramba 1 |	55	6	(10.9)	[4.110, 22.247]
Muramba 2#	53	6	(11.3)	[4.270, 23.028]
Rwakingi 1 A	54	0	(0)	[0, 6.603]
Rwakingi 1B	56	0	(0)	[0, 6.375]
Ibanda Parish				
Ibanda 1	58	1	(1.7)	[0.044, 9.236]
Ibanda 2	57	2	(3.5)	[0.428, 12.107]
Nyakabugha	57	4	(7.0)	[1.945, 17.004]
Kiharara	60	0	(0)	[0, 5.963]
Mihunga	52	0	(0)	[0, 6.848]
Mirimbo	53	5	(9.4)	[3.135, 20.659]
Ruboni	56	6	(10.7)	[4.035, 21.876]
Katooke Parish				
Katooke 1	66	4	(4.6)	[1.676, 14.797]
Katooke 2	56	5	(8.9)	[2.963, 19.619]
Kemihoko	56	2	(3.6)	[0.435, 12.313]
Kihindi	63	0	(0)	[0, 0.057]
Kinyangoye	54	6	(11.1)	[4.188, 22.631]
Mapata	60	3	(5.0)	[1.043, 13.924]
Mulehe	56	1	(1.8)	[0.045, 9.55259]
Nyangonge	59	1	(1.7)	[0.043, 9.086]
Kirongo	54	2	(3.7)	[0.451, 12.747]
Kibirizi Parish				
Bulindiguru	60	5	(8.5)	[2.761, 18.386]
Ihani	59	2	(3.4)	[0.413, 11.715]
Kasanzi	59	3	(5.1)	[1.061, 14.149]
Kabirizi	60	5	(8.3)	[2.761, 18.386]
Kikokera	55	2	(3.6)	[0.443, 12.526]
Muhambo Parish				
Bunyangoni	56	2	(3.6)	[0.43548, 12.313]
Katumba	58	2	(3.4)	[0.420, 11.908]
Maghoma	56	2	(3.6)	[0.435, 12.313]
Muhambo	59	1	(1.7)	[0.043, 9.086]
Nduguthu East	60	1	(1.7)	[0.042, 8.940]
Nduguthu West	58	0	(0)	[0, 0.062]
Mubuku Parish				
Izinga	58	5	(8.6)	[2.859, 18.982]

# indicates villages selected for phase 2

**Fig. 2:**
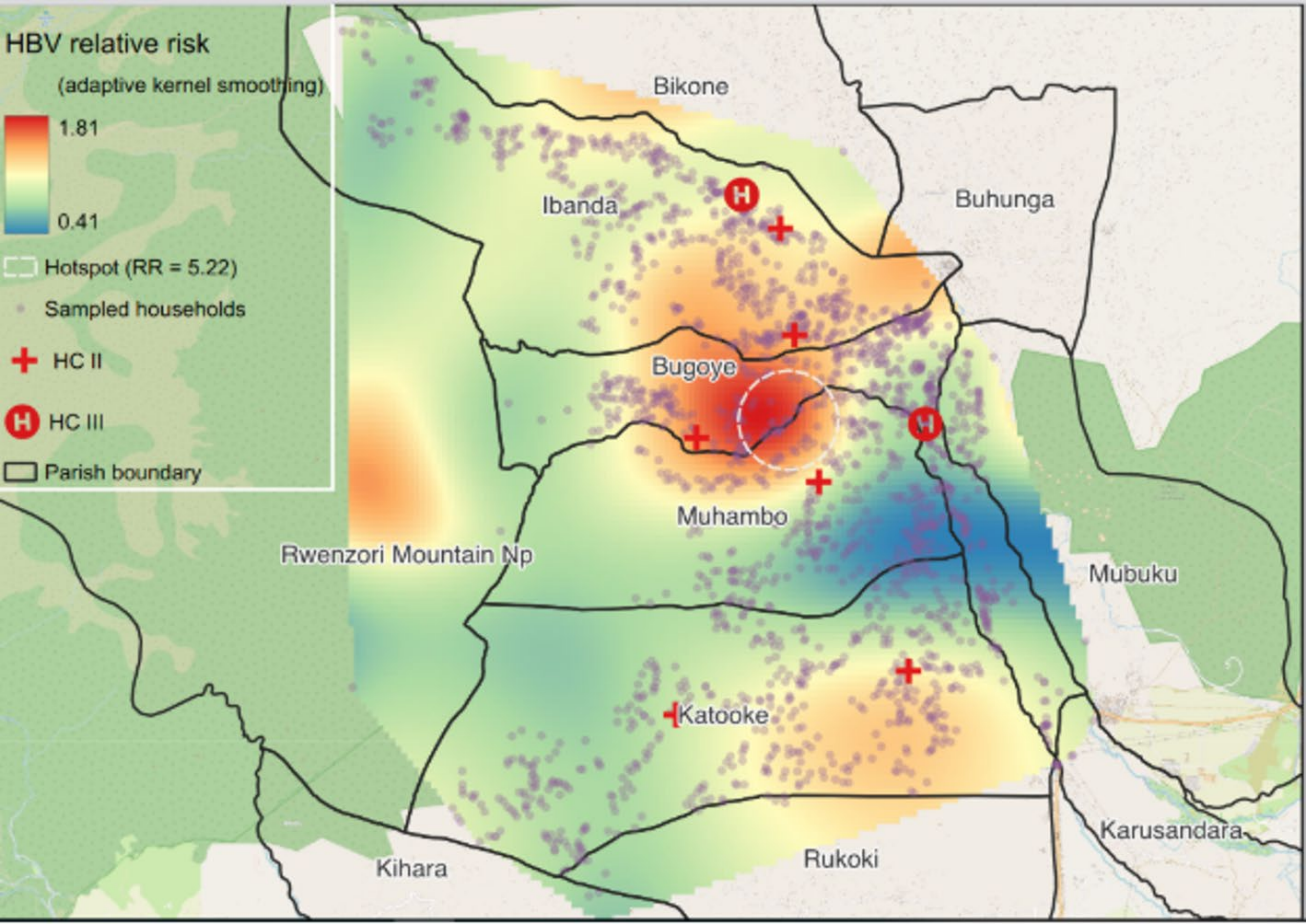
Map displaying the Hepatitis B prevalence of Bugoye Subcounty based on the household survey. The red/orange areas are the areas with relatively higher HBV risk compared to expected, whereas the blue/green areas are lower risk

The spatial distribution of HBsAg positivity showed a disproportionate HBV risk with the hotspot around the borders of Bugoye and Muhambo parishes, which had a relative risk of 5.2. Residents within this area were more than five times as likely to test positive for HBsAg compared with those outside the cluster. This hotspot lies between 4 health facilities and is characterised by rugged terrain. (Fig. 2).

In Phase 2, conducted from January 12 to 15, 2022, study staff visited 286 individuals from 74 households in Kanyamingho and Muramba 2 villages, including 50.3% (144/286) from Muramba 2 and 49.7% (142/286) from Kanyamingho village. In total, we enrolled 125 individuals ≥ 15 years of age and 161 children < 15 years of age.

Participants in Muramba 2 reported higher rates of HBV immunisation than those in Kanyamingho (66.6% vs. 40.4%, respectively). Additionally, participants in Muramba 2 reported the following: greater travel time to a health facility (30 min vs. 60 min), a greater proportion of home births (30 min vs. 60 min), more frequent sharing of sharps such as razors (71.4% vs. 8.1%), as well as modestly higher receipt of blood transfusions (7.9% vs. 1.6%) compared to those in Kanyanamigho (Table 2).

**Table 2:** Demographic, social-behavioural, clinical, and health system characteristics of the 125 individuals aged 15 and above years in Muramba 2 and Kanyamingho villages

Characteristic	Villages		*p*-Value
	Kanyamingho (*N* = 62) n (%)	Muramba 2 (*N* = 63) n (%)	
Age years (median (min – max))	32(16–86)	30.5(16–84)	
Age categories (years)			
16–24	19(30.7)	16(25.4)	0.880
25–34	17(27.4)	21(33.3)	
35–44	7(11.3)	7(11.1)	
≥ 45	19(30.7)	19(30.2)	
Sex of respondents			
Female	43(69.4)	49(77.8)	0.285
Male	19(30.6)	14(22.2)	
Marital status			
Widowed/divorced	10(16.1)	3(4.8)	**< 0.001**
Married	29(46.8)	50(79.3)	
Single	23(37.1)	10(15.9)	
Number of sexual partners			
None	2(3.3)	12(20.3)	0.029
one	48(80.0)	38(64.4)	
Two or more	10(16.7)	9(15.3)	
Religion			
Anglican	21(34.4)	42(66.7)	**< 0.001**
Catholic	33(54.1)	21(33.3)	
Others	7(11.5)	0(0.0)	
Occupation			
Unemployed	12(21.1)	13(20.6)	**0.011**
Employed	12(21.1)	4(6.4)	
Farmer	33(57.8)	46(73.0)	
Education level			
No formal education	8(12.9)	10(15.9)	0.226
Primary	25(40.3)	33(52.4)	
Secondary	29(46.8)	20(31.7)	
Average family income (ugx)			
< 100,000	21(33.9)	32(50.8)	0.003
100,000–299,999	23(37.1)	27(42.9)	
300,000–500,000	18(29.0)	4(6.3)	
Family size			
3–5 members	33(53.2)	31(50.8)	0.079
6–9 members	18(29.0)	26(42.6)	
10–13 members	11(17.8)	4(6.6)	
Share cutting equipment			
No	57(91.9)	18(28.6)	**< 0.001**
Yes	5(8.1)	45(71.4)	
Hepatitis B status			
Negative	62(100.0)	57(90.5)	**0.013**
Positive	0(0.0)	6(9.5)	
Ever tested for Hepatitis B			
No	32(51.6)	18(28.6)	**0.009**
Yes	30(48.4)	45(71.4)	
Immunized against Hepatitis B			
No	34(54.8)	19(30.2)	**0.013**
Yes	25(40.4)	42(66.6)	
Don’t know	3(4.8)	2(3.2)	
Ever had positive HIV test			
No	62(100)	58(93.5)	0.163
Yes	0(0.0)	1(1.7)	
Don’t know	0(0.0)	3(4.8)	
Ever received blood transfusion			
No	60(98.4)	58(92.1)	0.158
Yes	1(1.6)	5(7.9)	
Receipt of a parenteral medication			
No	40(64.5)	50(79.4)	0.065
Yes	22(35.5)	13(20.6)	
Place of birth			
Home	24(39.3)	44(69.8)	**0.005**
Health facility	34(55.8)	18(28.6)	
Don’t know	3(4.9)	1(1.6)	
Born by cesarean section			
No	56(90.3)	62(98.4)	0.138
Yes	1(1.6)	0(0.0)	
Don’t know	5(8.1)	1(1.6)	
Time to Health facility			
<=30 min	56(90.3)	15(23.8)	**< 0.001**
31–60 min	6(9.7)	32(50.8)	
> 60 min	0(0.0)	16(25.4)	

None of the participants aged < 15 years tested positive for HbsAg in either village. Among the 125 participants aged ≥ 15 years in both villages, 4.8% (*n* = 6) tested positive, all of whom resided in Muramba 2. Notably, all HBsAg-positive participants were married females who reported working as subsistence farmers. Among these, the majority, 83.3% (5/6), were aged 25 to 34 years old with limited educational attainment and no prior testing for HBV.

Descriptive comparisons suggested that HBsAg positivity was more common among participants who reported previous HBV testing, a history of blood transfusion, and receipt of parenteral medications. Higher proportions of HBsAg positivity were also observed among participants who reported sharing sharp instruments and among those residing more than 60 min from a health facility. (Table 3).

**Table 3:** Distribution of HBsAg positivity by demographic, social-behavioural, clinical, and health system characteristics of the 125 individuals aged 15 years and above in Muramba 2 and Kanyamingho villages

Characteristic	Hepatitis BsAg positivity	
	Negative *n* (%) 119(95.2%)	Positive *n* (%) 6(4.8%)
Village		
Muramba	57(90.5%)	6(9.5%)
Kanyamingho	62(100.0%)	0(0.0%)
Age categories (years)		
15–24	35 (100.0)	0 (0.0)
25–34	33 (86.8)	5 (13.2)
35–44	14 (100.0)	0 (0.0)
≥ 45	37 (97.4)	1 (2.6)
Sex of respondents		
Female	86 (93.5)	6 (6.5)
Male	33 (100.0)	0 (0.0)
Marital status		
Widowed/divorced	13 (100.0)	0 (0.0)
Married	73 (92.4)	6 (7.59)
Single	33 (100)	0 (0.0)
Number of sexual partners		
None	11 (78.6)	3 (21.4)
One	84 (97.7)	2 (2.33)
Two or more	18 (95.0)	1 (5.3)
Religion		
Anglican	60 (95.2)	3 (4.8)
Catholic	51 (94.4)	3 (5.6)
Others	7 (95.2)	0 (0.0)
Occupation		
Unemployed	25 (100.0)	0 (0.0)
Employed	16 (100.0)	0 (0.0)
Farmer	73 (92.4)	6 (7.6)
Education level		
No formal education	18 (100.0)	0 (0.0)
Primary	53 (91.4)	5 (8.6)
Secondary	48 (98.0)	1 (2.0)
Average family income (ugx)		
< 100,000	49 (92.4)	4 (7.6)
100,000–299,999	48 (96.0)	2 (4.0)
300,000–500,000	22 (100.0)	0 (0.0)
Family size		
3–5 members	60 (93.8)	4 (6.3)
6–9 members	42 (95.5)	2 (4.6)
10–13 members	15 (100.0)	0 (0.0)
Ever tested for Hepatitis B		
No	49 (98.0)	1 (2.0)
Yes	70 (93.3	5 (6.7)
Immunized against Hepatitis B		
No	50 (94.3)	3 (5.7)
Yes	64 (95.5)	3 (4.5)
Don’t know	5 (100.0)	0 (0.0)
Ever had positive HIV test		
No	114 (95.0)	6 (5.0)
Yes	1 (100.0)	0 (0.0)
Don’t know	3 (100.0)	0 (0.0)
Ever received blood transfusion		
No	113 (95.8)	5 (4.24)
Yes	5 (83.3)	1 (16.7)
Receipt of parenteral medications		
No	88 (97.8)	2 (2.2)
Yes	31 (88.6)	4 (11.4)
Place of birth		
Home	64 (94.1)	4 (5.9)
Health facility	50 (96.1)	2 (3.9)
Don’t know	4 (100.0)	0 (0.0)
Born by caesarean		
No	112 (94.9)	6 (5.1)
Yes	1 (100.0)	0 (0.0)
Don’t know	6 (100.0)	0 (0.0)
Share sharp equipment		
No	73 (97.3)	2 (2.7)
Yes	46 (92.0)	4 (8.0)
Time to Health facility		
<=30 min	70 (98.6)	1 (1.4)
31–60 min	36 (94.7)	2 (5,3)
> 60 min	13 (81.2)	3 (18.8)

## Discussion

In a cross-sectional survey conducted in rural western Uganda, we found that while the seroprevalence of HBsAg remains relatively high among adults (4.5%) [33–35], there is marked heterogeneity in risk, both by age category and geography, even over a relatively small area. Notably, we did not identify any infections in 161 children who were tested. Instead, HBsAg-positivity was more common among relatively young (age 25–34) women. Moreover, in the village-level descriptive analysis, HBV infection was higher in those with prior receipt of intravenous medications and blood transfusions, which may suggest iatrogenic drivers of infection, but it requires further investigation with a more rigorous study design.

In regard to geographic risk, we found that HBsAg prevalence was concentrated in a single river valley in the central part of the sub-county. This is an area characterised by rugged, highland terrain, yet nearly equidistant between four health centres. Although the hotspot was located approximately equidistant from four health facilities, the challenging terrain may limit the practical accessibility of preventive and curative health services. This finding suggests that geographic proximity to health facilities alone may not be sufficient to ensure access to services such as childhood immunisation, antenatal care, and HBV screening. Alternatively, the observed clustering may reflect localised social, behavioural, or household-level factors that facilitate transmission within the community [36]. This is in line with prior studies that have shown that limited geographic accessibility is a very important factor in HBV infection control [37]. When we further explored the two villages in the follow-up phase, we found that participants from Muramba, who were more likely to walk for more than an hour to the nearest health facility, were also more likely to deliver at home and had the highest prevalence of HBV in the subcounty. Interestingly, HBsAg positivity did not appear to differ by vaccination status; however, Kanyamingho had lower vaccination and lower prevalence, in contrast to prior studies in northern Uganda that have documented a higher prevalence of hepatitis B in the unvaccinated population [38, 39].

While our findings are suggestive of iatrogenic transmission, the small number of cases precludes definitive conclusions. These associations should be viewed as hypothesis-generating. We observed a higher frequency of HBsAg positivity among participants reporting a history of intravenous medication administration or blood transfusion. This aligns with findings from prior studies that found blood transfusion history to be a significant risk factor for HBV [40]. These findings suggest that iatrogenic transmission may be an important but underrecognized driver of HBV infection. While in our experience, re-use of needles and other equipment used to administer intravenous medications and blood products is not common in the public sector, much less is known about the private sector, where economic concerns may drive poor clinical practices. Specifically, we highlight our prior work examining the role of private-sector drug shops in the sub-county [41]. In that study, we found that intravenous antimalarial medications such as artesunate were frequently administered – typically by vendors with minimal medical training – in drug shops. Given that the highest prevalence villages were located in areas with limited access to health facilities and thus may disproportionately utilise private drug shops, there is an urgent need to examine the role these shops may play in blood-borne pathogen transmission. However, as this was a descriptive and exploratory study with a small number of positive cases, these findings should not be interpreted as evidence of a strong causal relationship and require further investigation in larger studies.

Notably, we did not identify any HBV infections among children in either village. Although the number of children tested was limited, and the study design could not allow definitive conclusions, our findings align with another study in northern Uganda which evaluated the impact of the pentavalent HBV vaccine in northern Uganda in which the HBV prevalence was found to increase with age and peaked within the 20–39 years age group; that study noted a prevalence of 0% among those below 5 years, 14.9% in the 15–19 year age group and 13.7% in the 20–39 year age group [39]. Our findings are consistent with another study that found mother-to-child and early life transmission were infrequent [38]. One possible explanation could be that the children below 15 years were born after the introduction of HBV vaccination into the Uganda national immunisation program in 2002 and may have therefore benefited from vaccination [42]. This suggests that HBV infection is likely to happen later in life via horizontal transmission. It also supports the current immunisation strategy and the need for HBV vaccination before 6 weeks [38].

## Strengths and limitations

Our study has several strengths, including the incorporation of GIS data to inform our interpretation of results and the use of both a population‑based survey and a follow-up cross‑sectional assessment of two purposively selected villages representing high‑ and low‑prevalence settings. The study also has limitations, the foremost of which was the relatively small sample size and the small number of positive individuals (*n* = 6). The small number of HBsAg‑positive participants identified in Phase 2 limits the precision of our estimates and restricts the strength of inferences that can be drawn from results. This small number also precluded logistic regression analyses; future larger studies should confirm these associations. However, we are reassured by the rigour of our sampling approaches and seroprevalence estimates that are similar to UPHIA, the most rigorous study of HBV in Uganda to date. Secondly, many of our variables were self-reported and thus may have been prone to reporting or social desirability bias, specifically on topics such as sexual partners, prior testing, and vaccination status. Additionally, Vaccination status was based on participant or caregiver recall rather than verification from vaccination cards or health facility records, introducing the potential for misclassification bias. Lastly, participants were enrolled in Phase 2 at a time when schools had just opened following COVID-19 lockdowns, which may have affected who was present in the home during study visits.

These findings may be generalizable to other rural, highland areas in East Africa with similar geography and healthcare access patterns.

## Conclusions

The seroprevalence of HBsAg in Bugoye subcounty was relatively high but marked by substantial geographic and demographic variability. The absence of infection in children is consistent with the hypothesis that vaccination programs have been effective, though this observational finding requires confirmation. We observed associations between HBsAg positivity and a history of intravenous medication administration and blood transfusion. These findings suggest the need for further investigation into potential healthcare-related transmission pathways, particularly in private-sector settings where infection prevention and control practices may vary. Given the small number of HBsAg-positive cases, these associations should be interpreted cautiously. Future studies are needed to better understand the role of healthcare-related exposures in HBV transmission and to inform targeted interventions. Potential interventions include strengthening the district health office oversight of private drug shops, improving infection prevention and control practices, safe injection and sterilisation procedures, adherence to blood screening guidelines, and targeted education for healthcare workers and communities on HBV transmission, prevention, testing, and vaccination.

## Data Availability

The datasets used and/or analysed during the study will be available from the corresponding author on reasonable request **.** The deidentified individual data that supports the results will be shared beginning 9 to 36 months following publication, provided the investigator who proposes to use the data has approval from an Institutional Review Board (IRB), Independent Ethics Committee (IEC), or Research Ethics Board (REB), as applicable, and executes a data use/sharing agreement with UNC.
